# Frequency Stability Prediction of Power Systems Using Vision Transformer and Copula Entropy

**DOI:** 10.3390/e24081165

**Published:** 2022-08-21

**Authors:** Peili Liu, Song Han, Na Rong, Junqiu Fan

**Affiliations:** 1 Department of Electrical Engineering, Guizhou University, Guiyang 550025, China; 2 Guian Company Guizhou Power Grid, Guiyang 550003, China

**Keywords:** frequency stability prediction, vision transformer, copula entropy, deep learning, power system

## Abstract

This paper addresses the problem of frequency stability prediction (FSP) following active power disturbances in power systems by proposing a vision transformer (ViT) method that predicts frequency stability in real time. The core idea of the FSP approach employing the ViT is to use the time-series data of power system operations as ViT inputs to perform FSP accurately and quickly so that operators can decide frequency control actions, minimizing the losses caused by incidents. Additionally, due to the high-dimensional and redundant input data of the power system and the O(N^2^) computational complexity of the transformer, feature selection based on copula entropy (CE) is used to construct image-like data with fixed dimensions from power system operation data and remove redundant information. Moreover, no previous FSP study has taken safety margins into consideration, which may threaten the secure operation of power systems. Therefore, a frequency security index (FSI) is used to form the sample labels, which are categorized as “insecurity”, “relative security”, and “absolute security”. Finally, various case studies are carried out on a modified New England 39-bus system and a modified ACTIVSg500 system for projected 0% to 40% nonsynchronous system penetration levels. The simulation results demonstrate that the proposed method achieves state-of-the-art (SOTA) performance on normal, noisy, and incomplete datasets in comparison with eight machine-learning methods.

## 1. Introduction

To respond to the global environmental crisis, 137 countries agreed to achieve carbon neutrality by approximately 2050 after COP26 [1]. As an essential part of this climate action plan, the large-scale replacement of fuel resources with renewable resources will be able to effectively reduce carbon emissions [2]. Nevertheless, grid frequency support becomes weakened as large-scale renewable generators are connected, which is a challenging issue for power systems that must accommodate renewable energy penetration [3,4]. Specifically, fuel resources in power systems usually belong to synchronous generators that provide inertia and primary operating reserves to maintain system frequency stability [5]. However, the inertia that maintains the system frequency stability is declining because synchronous generators are being replaced by nonsynchronous generators (renewable resources) [6]. To arrest and stabilize frequency volatility in renewable-energy-penetrated power systems, it is essential to accurately and quickly predict system frequency stability, which helps system planners and dispatchers to determine the corresponding control measures in advance, such as under frequency load shedding [7,8], frequency regulation using renewable-power production units [9], frequency regulation using loads [10], and frequency regulation using storage devices [11].

The traditional methods for frequency stability prediction (FSP) are model-driven methods, including the time-domain simulation (TDS) approach and its equivalent models, which are rigorous and logistic in derivation. TDS, as the cornerstone of this research field, provides the most accurate frequency response results via high-order nonlinear equations and stepwise integration. Nevertheless, TDS is not available for online FSP because this method takes much time for its computations. Equivalent model-based methods have been proposed to reduce the time consumption for FSP. However, these equivalent methods achieve increased computing efficiency by simplifying their generator models, resulting in accuracy decreases. Furthermore, the benefit derived from the development of data-driven methods, such as machine learning (ML) methods [12,13], increases the feasibility of online FSP in various scenarios, which fills the research gap mentioned above.

As the main branch of ML methods, deep-learning (DL) methods achieve vigorous performance in online FSP due to their powerful nonlinear modeling capabilities. For instance, transformers in DL have strong representation capabilities; they can extract global features from the time-series data of power system operations. However, their O(N^2^) computational complexity imposes high computational and time costs. In particular, the high-dimensional and redundant data obtained from large-scale power systems make the success of the transformer costly. Feature selection based on copula entropy (CE) is a simple and effective way to ease the computational burden caused by a transformer and remove redundant information. Given the advantages of transformers and CE, this paper uses these approaches to form a DL framework and applies it to accurately and quickly predict frequency stability, where the aim is to achieve the best performance without massive computational resources. Additionally, to fully consider frequency response characteristics, a frequency security index (FSI) is used as the prediction indicator of the DL methods.

The remainder of this paper is organized as follows: Section 2 introduces the related studies. Section 3 presents the ViT-based FSP method. The overall process of the proposed method is presented in Section 4. In Section 5, case studies are provided. In Section 6, the proposed method is discussed. Finally, Section 7 is devoted to conclusions and future work.

## 2. Related Work

This section provides a literature review regarding power system stability prediction, including model-driven and data-driven methods. In addition, the transformer models in DL, feature selection methods, and FSIs are also reviewed in this section.

### 2.1. Model-Driven Methods

The traditional model-driven methods are mainly divided into two types: (1) TDS and (2) its equivalent models. TDS involves power flow models [14,15] and component models [16,17], such as generators, turbines, boilers, governors, exciters, and power system stabilizers. According to its detailed simulation models, TDS can accurately depict dynamic frequency processes, but it is not able to perform online prediction in a power system due to its high computational burden. The equivalent model-based methods neglect the power flow models and simplify the component models to predict dynamic frequency responses; these models include the average system frequency [18,19] model and system frequency response (SFR) [20,21] model. In [20], a low-order SFR model was able to reduce the computational burden and limit the errors induced by frequency response estimation. In [21], an integration method was proposed to combine the SFR model and Type-3 wind turbines for dynamic frequency analysis. In [22], an improved SFR model was proposed to analyze the influences of thermal states on dynamic frequency responses by extending the typical SFR model with a thermodynamic boiler submodel. Overall, there is always a trade-off between computational efficiency and accuracy in the equivalent model-based methods.

### 2.2. Data-Driven Methods

Currently, power system operation data in real time can be accessed by wide-area measurement systems (WAMSs) with phasor measurement units (PMUs) [23]. Therefore, ML has been a popular technique for analyzing power system stability in recent years [24,25]. Various DL methods, such as long short-term memory (LSTM) [26], convolutional neural networks (CNNs) [27], and graph neural networks (GNNs) [28], have been widely applied to power system stability prediction. To the best of the authors’ knowledge, LSTM is good at extracting sequence features [29], CNNs are good at extracting local features [30], and GNNs are good at extracting topology structure features [31]. Each DL method mentioned above has corresponding advantages. Therefore, based on these characteristics, some scholars have developed multi-model combinations, such as CNN-LSTM [32], CNN-GRU [33], and GNN-LSTM [34] models, to further mine data from various aspects of information for prediction purposes. It is noteworthy that none of the former studies focused on transformer models in the research field of power system stability.

### 2.3. Transformer Models in DL

The original transformer, as a powerful DL model, was first proposed in [35]. Unlike a CNN or LSTM, a transformer can extract global features via an attention mechanism. The most representative work is the bidirectional encoder representations from transformers (BERT) [36]. At present, most state-of-the-art (SOTA) natural language processing (NLP) tasks involve BERT. Inspired by the stunning performance of transformers in NLP, the Brain Team of Google Research proposed a vision transformer (ViT) [37] model to solve computer vision (CV) tasks, and the ViT outperformed ResNet [38] in image classification. After this, researchers studying other CV tasks tried to use the transformer architecture as their backbone networks to achieve object detection [39] and semantic segmentation [40]. Inspired by the fantastic performance of transformers in NLP and CV, this paper first proposes a ViT-based FSP method to fill the research gap regarding the use of transformers in power system stability prediction.

### 2.4. Feature Selection Methods

The current feature selection approaches can be classified into three categories: (1) embedded methods; (2) filter-based methods; and (3) wrapper-based methods. Specifically, an embedded method is injected into the learning process of a forecasting model. A filter-based method is independent of any prediction model. A wrapper-based method is based on an optimization algorithm and a forecasting model [41]. In terms of this study, DL methods can automatically perform feature extraction, but at a high cost. We hope that there is a simple and effective way to remove apparently redundant features. CE-based feature selection is a filter-based method, so it can meet the above requirements. Compared with embedded and wrapper-based methods, filter-based methods have higher execution efficiency and greater generalization capabilities [42]. In particular, mutual information (MI) [43] and RReliefF are typical filter-based methods. In [44], CE is proven to be equivalent to MI but has a lower computational burden than MI.

### 2.5. Frequency Security Indices

Frequency prediction indicators can be divided into two kinds: frequency curve prediction [32,45] and frequency characteristics prediction [46,47]. However, the above frequency indicators only distinguish between frequency security and insecurity. None of the previous studies paid attention to safety margins. Delkhosh and Seifi [48] proposed an FSI considering all frequency key characteristics. Due to the decline in power system inertia, this paper combines the center-of-inertia frequency (COIF) and FSI to divide the system frequency responses into three categories, i.e., insecurity, relative security, and absolute security. Note that relative security reflects the safety margin of the frequency response, which can help operators avoid the risk caused by low inertia. For the above reasons, the FSI is used as the prediction indicator of the DL methods.

### 2.6. Our Contributions

Finally, the main contributions of this paper are summarized as follows.

- This paper proposes a ViT-based FSP method that predicts frequency security online following a disturbance.
- A CE-based feature selection method is used to construct image-like data with fixed dimensions, which can decrease the computational burden of the proposed model by removing redundant information.
- This paper develops a novel FSI as the predicted result of the model, which considers the safety margin and comprehensive characteristics of frequency compared with the traditional indicators.
- Case studies are conducted on a modified IEEE 39-bus system and a modified ACTIVSg500 system for projected 0% to 40% nonsynchronous system penetration levels, aiming to validate the proposed method’s efficacy and scalability.

## 3. ViT-Based FSP Method

### 3.1. Vision Transformer (ViT)

#### 3.1.1. Multihead Self-Attention

Normal qkv self-attention (SA) [49] is a building block for DL, and it is given by Equation (1). We compute a weighted sum over all values for every element in an input sequence $z\in {\mathbb{R}}^{N\times D}$. The attention weights *A_ij_* are based on the pairwise similarity between two elements in the sequence and their respective query *q^i^* and key *k^j^* representations.

(1) $$\left\{\begin{array}{cc} {}[q,k,v]={\mathrm{zU}}_{qkv} & U_{qkv}\in {\mathbb{R}}^{D\times 3D_{h}},\\ A=\mathrm{softmax}({\mathrm{qk}}^{T}/\sqrt{D_{h}}) & A\in {\mathbb{R}}^{N\times N},\\ \mathrm{SA}(z)=Av. & \end{array}\right.,$$

**Figure 1:**
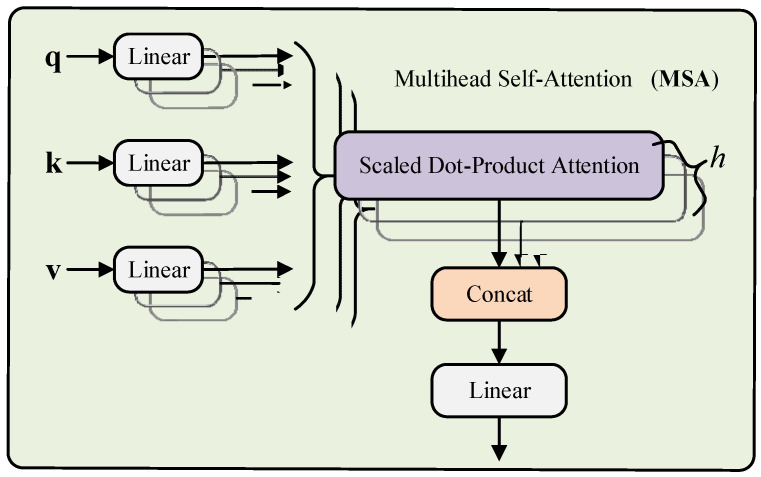
Multihead self-attention.

Figure 1 introduces a multihead self-attention (MSA) [35] mechanism that can be used to increase the performance of the SA layer, in which we run *h* self-attention operations (named “heads”) in parallel and project their concatenated outputs. It is given by Equation (2), and when changing *h*, *D_h_* is usually set to *D/h* to maintain the number of calculated parameters constant.

(2) $$\begin{array}{cc} {\mathrm{MSA}=[\mathrm{SA}}_{1}(z{);\mathrm{SA}}_{2}(z);\cdots {;\mathrm{SA}}_{h}{(z)]U}_{msa} & U_{msa}\in {\mathbb{R}}^{h\cdot D_{h}\times D} \end{array},$$

#### 3.1.2. ViT

**Figure 2:**
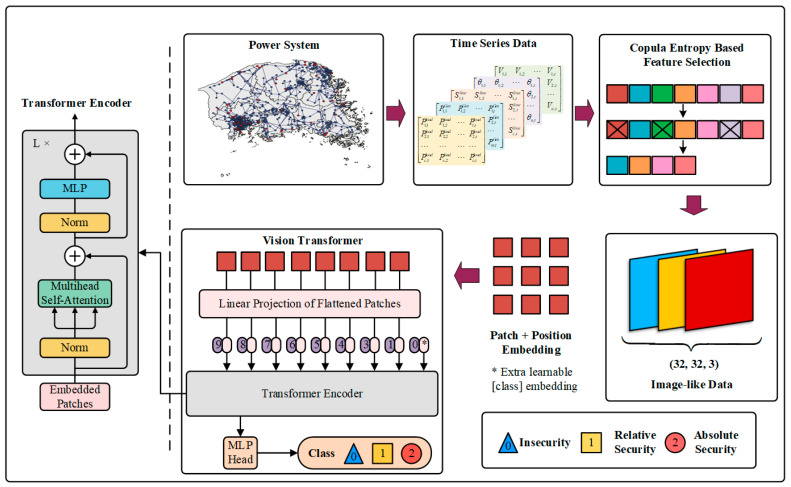
Framework of frequency stability prediction using Vision Transformer and Copula Entropy. (* denotes Position Embedding.)

The ViT adopts a pure transformer architecture, which has minimal changes for performing image classification tasks and achieves better performance than ResNet [37]. It follows the raw design of transformers as much as possible. Figure 2 depicts the framework of the ViT.

To process 2D images, an image $x\in {\mathbb{R}}^{H\times W\times C}$ is reshaped into *N* nonoverlapping image patches $x_{p}\in {\mathbb{R}}^{N\times (P^{2}\cdot C)}$ such that $p^{2}$ is the resolution of each image patch, *C* is the number of channels, and (*H*, *W*) is the resolution of the original image. The ViT performs a trainable linear projection that maps each vectorized path to 1D tokens $z_{i}\in {\mathbb{R}}^{d}$. The sequence of 1D tokens input into the subsequent transformer encoder is as follows:

(3) $$z=\left[Ex_{1},Ex_{2},\ldots ,Ex_{N}\right]+P_{1d},$$

where E denotes a linear projection that is equivalent to a 2D convolution [50], and *P*_1*d*_ denotes 1D position embeddings that are added to the patch embeddings to retain positional information. The tokens are passed through an encoder consisting of a sequence of transformer layers. Each layer ℓ comprises layer normalization (LN) [51], multilayer perception (MLP) [36], and MSA blocks, as follows:

(4) $${y=\mathrm{MSA}(\mathrm{LN}(z}^{\ell }{))+z}^{\ell },$$

(5) $$z^{\ell +1}={\mathrm{MLP}(\mathrm{LN}(y}^{\ell }{))+y}^{\ell },$$

The MLP is made up of two linear projections split by a GELU activation function [37], and the token dimensionality remains constant throughout all layers. Finally, a linear classifier is utilized to classify the encoded input.

As shown in Figure 2, power system operation data are reshaped into an equivalent form ${\mathbb{R}}^{H\times W\times C}$, where *H* is the sampling time series of the sensors, *W* is the dimensionality of the data, and *C* is the number of channels. Given such a transformation, FSP of power systems can also be carried out by the ViT model.

### 3.2. CE-Based Feature Selection

Statistical independence is a fundamental concept in the fields of statistics and ML. Copulas provide theoretical tools for uniformly representing the statistical associations between random variables [52]. The core of copula is the Sklar theorem [53], which shows that a multivariate density function can be denoted as a product of its marginal and copula density functions, indicating a dependence structure among the associated random variables.

Suppose that X represents random variables whose marginals and copula density are *u* and *c*(*u*), respectively. According to the copula density, the CE of X can be defined as follows:

(6) $$H_{c}(X)=-\int_{u}c(u)\log c(u)du,$$

where $c(u)=\frac{d^{N}C(u)}{du_{1}du_{2}\cdots du_{N}}$.

In [44], a parameter-free CE estimation approach was proposed, including two steps:

(1) Estimating the empirical copula density (ECD)
(2) Estimating the CE

For step 1, if independent identically distributed samples $\left\{x_{1},\ldots ,x_{T}\right\}$ are generated from random variables $X=\{x_{1},\ldots ,x_{N}{\}}^{T}$, one can easily estimate the ECD as follows:

(7) $$F_{i}(x_{i})=\frac{1}{T}\sum_{t=1}^{T}\chi (x_{t}^{i}\leq x_{i}),$$

where *i* = 1,..., and *N* and *χ* represent an indicator function. Let $u=[F_{1},\ldots ,F_{N}]$; then, one can derive a new sample set $\left\{u_{1},\ldots ,u_{T}\right\}$ as data from the ECD *c*(u).

Once the ECD is estimated, step 2 is essentially an entropy estimation problem. The k-nearest-neighbor method [43] is utilized to estimate the CE. A larger CE denotes a stronger correlation between the tested variables. The desired features can be obtained by measuring the CE values between the input features and the target features.

In this work, power system operation data are reshaped into three 32 × 32 dimensional matrices, i.e., image-like data with three channels and 32 pixels, via CE-based feature selection. In this process, considerable redundant information is removed from the power system operation data. Therefore, such input data with fixed dimensions are utilized as the inputs of the ViT to avoid an unnecessary computational burden.

### 3.3. Frequency Security Index

#### 3.3.1. Center-of-Inertia Frequency

The frequency of each generator fluctuates around the COIF when a sudden incident occurs in the power system. Therefore, the COIF is commonly used to represent the power system frequency in load shedding schemes [54]. The COIF is given by Equation (8).

(8) $$f_{COI}=\left(\sum_{i=1}^{N}H_{i}S_{i}f_{i}\right)/\left(\sum_{i=1}^{N}H_{i}S_{i}\right),$$

where *H_i_*, *S_i_*, and *f_i_* represent the inertia constant, rated apparent power, and frequency of generator *i*, respectively. *N* stands for the number of synchronous generators. In this work, the COIF is used to calculate the proposed FSI.

#### 3.3.2. Insecure Boundaries and Secure Boundaries

**Figure 3:**
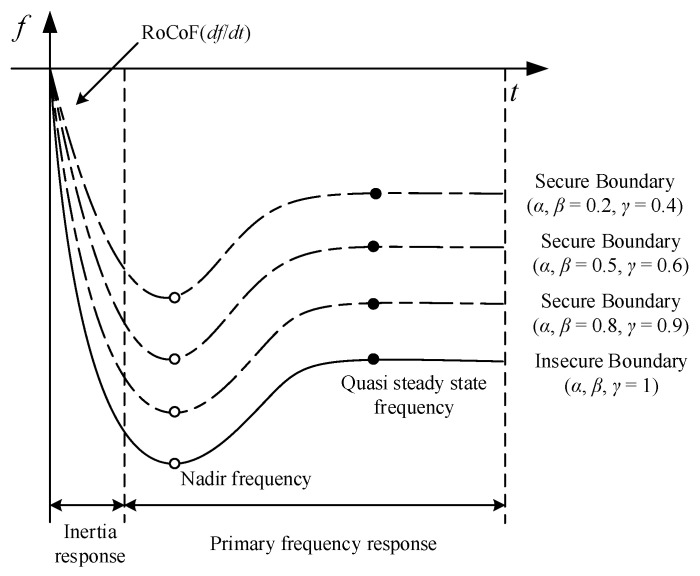
Impact of different security coefficients (*α*, *β*, and *γ*) on an SB.

**Insecure boundaries** (IBs) are provided by standards and policies to maintain the system stability and reliability, i.e., the maximum frequency deviation (FD), rate of change of frequency (RoCoF), and quasi-steady-state frequency deviation (QSSFD). As depicted in Figure 3, an IB is a constant boundary distinguishing between the secure (stable) and insecure (unstable) frequencies after an active power disturbance.

**Secure boundaries** (SBs) distinguish between absolute security and relative security. As depicted in Figure 3, an SB is a flexible boundary determined by the disturbance size, and different values of *α*, *β,* and *γ* lead to different SBs, where *α*, *β,* and *γ* are dependent on the disturbance size, as defined in Equations (9)–(12).

The detailed calculation process of the IB and SB is shown in Table 1. Δ*f_c_*, *RoCoF*, and Δ*f_s_* in Table 1 represent the FD, RoCoF, and QSSFD, respectively. Δ*f_c_*^max^, *RoCoF*^max^, and Δ*f_s_*^max^ in Table 1 represent the maximum FD, RoCoF, and QSSFD, respectively. *α*, *β*, and *γ* in Table 1 represent the security coefficients of the FD, RoCoF, and QSSFD, respectively. The security coefficients (*α*, *β,* and *γ*) are defined in Equations (9)–(12).

(9) $$\begin{array}{cc} k_{T}=\frac{T_{\max}-T_{\min}}{M_{\max}-M_{\min}} & \left(T=\alpha ,\beta ,\gamma \right) \end{array},$$

(10) $$\alpha =\left\{\begin{array}{ll} 0.2, & M\leq M_{\min},\\ k_{\alpha }\cdot M, & M_{\min}<M\leq M_{\max},\\ 0.8, & M_{\max}<M, \end{array}\right.,$$

(11) $$\beta =\left\{\begin{array}{ll} 0.2, & M\leq M_{\min},\\ k_{\beta }\cdot M, & M_{\min}<M\leq M_{\max},\\ 0.8, & M_{\max}<M, \end{array}\right.,$$

(12) $$\gamma =\left\{\begin{array}{ll} 0.4, & M\leq M_{\min},\\ k_{\gamma }\cdot M, & M_{\min}<M\leq M_{\max},\\ 0.9, & M_{\max}<M, \end{array}\right.,$$

where *M* represents the disturbance size; *M*_max_ and *M*_min_ respectively represent the maximum and minimum disturbance sizes, which are reference values determined by the historical disturbances in the power system; and *k*_T_ represents the linear coefficient of the three security coefficients.

#### 3.3.3. Calculation of the FSI

The proposed FSI aims to qualitatively evaluate the frequency stability of a power system for a specified operating condition. When an incident occurs, the system frequency response can be divided into three states: insecurity, relative security, and absolute security. In Equation (13), the numbers 0, 1, and 2 indicate insecurity, relative security, and absolute security, respectively.

(13) $$\begin{array}{c} SS(\varphi )=\left\{\begin{array}{ll} 2, & SB(\varphi_{i})<\varphi_{i},\\ 1, & IB(\varphi_{i})<\varphi_{i}\leq SB(\varphi_{i}),\\ 0, & \varphi_{i}\leq IB(\varphi_{i}), \end{array}\right.\\ \left(\varphi_{1,2,3}=\Delta f_{c},RoCoF,\Delta f_{s}\right) \end{array},$$

where *φ_i_* indicates three frequency characteristics, such as Δ*f_c_*, *RoCoF*, and Δ*f_s_*. *SB*(*φ*) and *IB*(*φ*) are presented in Table 1. Furthermore, the minimum value among the three frequency characteristics is the FSI, which is given by Equation (14).

(14) $$\mathrm{FSI}=\min\left\{\begin{array}{ccc} SS(\varphi_{1}), & SS(\varphi_{2}), & SS(\varphi_{3}) \end{array}\right\},$$

**Figure 4:**
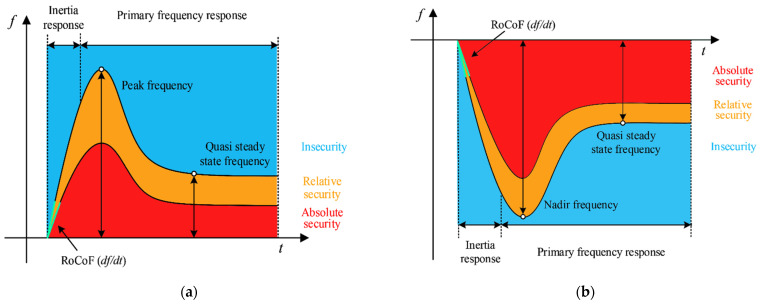
The effect diagram of the FSI for ΔP>0 and ΔP<0: the red zone denotes absolute security, the orange zone denotes relative security, and the blue zone denotes insecurity: (**a**) ΔP>0; (**b**) ΔP<0. ($\Delta {P=P}_{Gen}-P_{load}$ ).

For a clear understanding of the FSI, the effect diagram of the FSI is illustrated in Figure 4. If the COIF curve is located in the red zone, the system frequency is absolutely secure. If some parts of the COIF curve are located in the orange or blue area, the system frequency is relatively secure or insecure, respectively.

It is worth noting that the occurrence times of the maximum FD and the maximum QSSFD are not included in this paper. Due to their weak correlations with the disturbance size, it is not appropriate for the occurrence times of the maximum FD and the maximum QSSFD to undergo a similar process.

## 4. Overall Process of the Proposed Method

### 4.1. Raw Database

Original feature formation is critical for ensuring the accuracy of the FSP results. For a sudden disturbance in a power system, generators withstand the unbalanced power based on the corresponding synchronization factor. The synchronization factor between node *j* and node *k* is represented as:

(15) $$SP_{jk}=V_{j}V_{k}(B_{jk}\cos\delta_{jk}-G_{jk}\sin\delta_{jk}),$$

where *V* and *δ* denote the voltage amplitude and phase angle difference, respectively, and *B_jk_* and *G_jk_* denote the transfer impedance. Therefore, the voltage amplitude and phase angle of each bus [32] should be added to the original features. Furthermore, the power imbalance ΔP for a generator is defined as:

(16) $$\Delta P=P_{m}-P_{e}=2\frac{H}{f_{N}}\frac{df}{dt},$$

where *P_m_* and *P_e_* represent the mechanical power and electrical power of the generator, respectively. *H* stands for the inertia constant of the generator. *f_N_* stands for the system operational frequency. Referring to Equation (16), the electrical power values of the generators [32] are also selected as original input features. Note that the electrical power values of the nonsynchronous generators also might be related to frequency stability, according to [55,56,57]. Thus, in our work, the electrical power values of all generators are selected as original input features. Furthermore, the active power load of each bus and the apparent power of each line are also selected as original input features, as they can reflect the current power flow situation. In practice, sensors (i.e., PMUs) and TDS software (i.e., PSS/E, DIgSILENT) are able to provide the above data as a raw database. Specifically, they are listed in Table 2.

### 4.2. Offline Training

**Figure 5:**
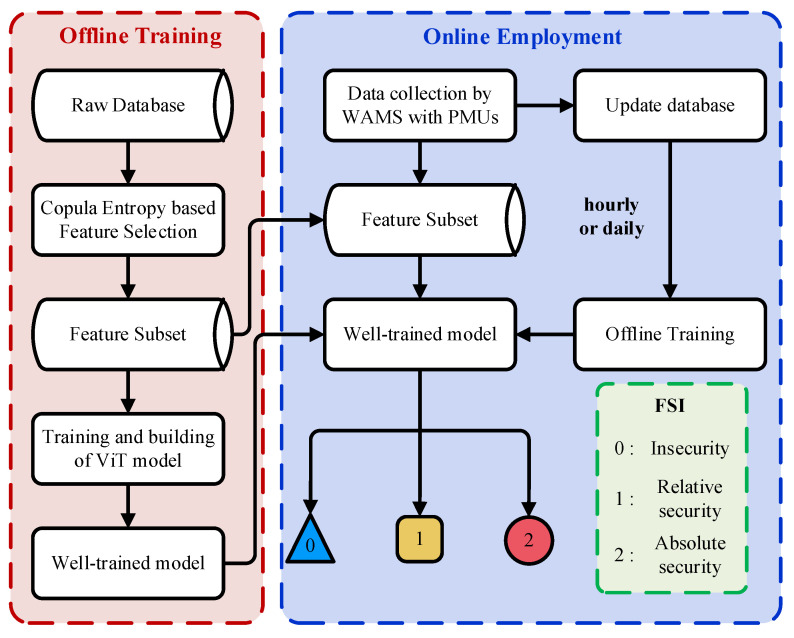
Flow chart for implementing the proposed method.

As illustrated in Figure 5, the offline training process of the proposed method includes two parts: (1) performing CE-based feature selection and (2) training and building the ViT model.

The first part calculates the CE values between the input data at the initial moment *t*_0_ and the FSIs. By sorting the CE values, the desired feature subset is obtained, the dimensionality of which is 96. Then, the data shape of the feature subset is reshaped into three channels, 32 features, and 32 sampling points, similar to an RGB image of 32 pixels. Moreover, zero-mean normalization [30] is used to eliminate the magnitude differences between different features before inputting them into the model, and this process is defined as follows:

(17) $$x^{*}=\frac{x-\mu }{\sigma },$$

where μ is the mean of all data, σ is the standard deviation of all data, and x^*^ is the normalized data.

For the second part, the loss function is the cross-entropy error function [30]. The optimization solver adopts Adam [58] to update parameters. The number of epochs is set to 200, and the batch size is set to 200 for each epoch. The initial learning rate is 0.0005, and the CosineAnnealingLR [59] schedule is used to decrease the learning rate to yield improved training efficiency.

### 4.3. Online Application

All steps of the online application are shown in Figure 5. The post-fault input data in the feature subset can be sampled by a WAMS with PMUs, and then these data input into the well-trained model, enabling the quick prediction of the FSIs, i.e., insecurity, relative security, and absolute security. The operators utilize corresponding controls and strategies by referring to the prediction results to minimize the loss caused by the incident. Moreover, note that the model only needs data from the feature subset in the online application stage, which means that the PMU does not cover the entire power system. Because PMUs are expensive, this can increase the economy of the ML system in online applications [60]. Finally, online updating of the database and retraining of the model can be carried out hourly or daily to adapt to various system operation situations [27].

### 4.4. Evaluation Indicators

In this paper, the FSP of power systems is a classification task. Therefore, accuracy (*ACC*), precision (*PRE*), recall (*REC*), and F-measure (*F*1) are employed as the evaluation metrics. These metrics are defined in Equation (18).

(18) $$\left\{\begin{array}{l} ACC=\sum_{i=0}^{2}TP_{i}/n_{total}\\ PRE_{i}=TP_{i}/\left(TP_{i}+FP_{i}\right)\\ REC_{i}=TP_{i}/\left(TP_{i}+FN_{i}\right)\\ F1=2*PRE*REC/(PRE+REC) \end{array}\right.,$$

where *TP_i_*, *FP_i_*, and *FN_i_* are the number of true-positive samples, the number of false-positive samples, and the number of false-negative samples under each security state *i*, respectively. *n_total_* is the total number of samples. *PRE_i_*, and *REC_i_* are the precision and recall under each security state *i*, respectively, whose average values are *PRE* and *REC*.

### 4.5. Equipment and Software

All tested algorithms are implemented in Pytorch-v1.10.1 and Scikit-learn-v1.0.2. The CE-based feature selection process is provided by pycopent-v0.2.3, which is available in R or Python. Moreover, all algorithms are trained on a personal computer with an Intel Core(TM) i5-12600KF CPU @ 3.70 GHz (Santa Clara, CA, USA), 32 GB of RAM and an RTX 3060 GPU ((Santa Clara, CA, USA)).

## 5. Case Studies

### 5.1. A Modified New England 39-Bus System

Numerical simulations were implemented on a modified New England 39-bus system with PSS/E [61] to simulate the data acquired by a WAMS and PMUs. To approximate the system behavior [27], the load levels of the power system were set to 50%, 51%, …, and 100% of the original load levels [32]. The same ratio was also used to scale the generation power, but extra modifications were provided to ensure that all input data fall within a reasonable range [27]. Under different power flow levels, the sudden load volatility was set to the active power disturbance [32]. The disturbance was assumed to occur on all load buses. The fault sizes were set to range from −500 MW to 500 MW at intervals of 100 MW. The fault occurrence time was set at the simulation start moment (0 s), the simulation duration was 60 s, and the sample rate was 100 Hz for each incident. Additionally, wind farms were connected to bus 2, bus 29, and bus 39 of the system. Dynamic wind farm models were provided by the Western Electricity Coordinating Council (WECC) [62]. Specifically, the wind turbine converter module adopted WT3G2, the electrical control module adopted WT3E1, the mechanical control module adopted WT3T1, and the pitch control module adopted WT3P1. The detailed parameters of dynamic wind farm models are listed in Appendix A. Changing the power output of the generating units controlled the renewable energy penetration rate (REPR). This study set the REPR to 0%, 5%, …, and 40% of the total generation power output.

The detailed configurations utilized for dataset generation are summarized in Table 3. The FSI was used for each sample to annotate the corresponding frequency stability category. The required input data for the FSI calculation process are listed in Table 4. Consequently, 69,768 labeled samples were formed under the above conditions. As described in the CE-based feature selection discussion, the input data obtained within 0.32 s were selected as a subset with 96 features. The number of sample points was T=0.32/0.01=32, and the input sample of each disturbance was $X\in {\mathbb{R}}^{32\times 32\times 3}$. In the subsequent experiments, the dataset was divided randomly at a 7:3 ratio into training and test datasets to assess the performance discrepancies among various methods.

**Figure 6:**
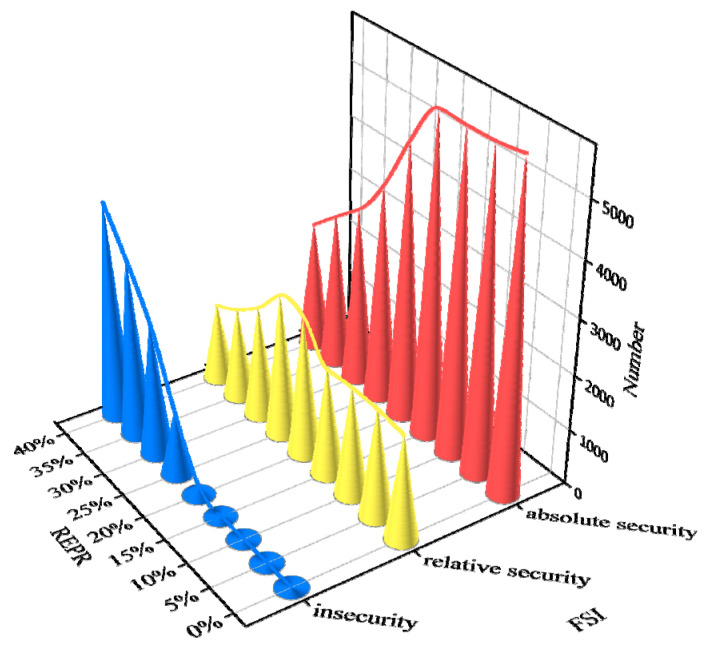
FSI distributions in the modified New England 39-bus system under different REPRs. The *x*-axis represents the frequency security index (FSI), i.e., insecurity, relative security, and absolute security. The *y*-axis represents the renewable energy penetration rate (REPR). The *z*-axis represents the number of each kind of sample among the FSIs.

In this work, the sample annotation process was based on the FSI. Each sample belonged to one of three frequency categories, i.e., insecurity, relative security, and absolute security. To observe the correlation between the REPR and FSI, Figure 6 presents the distribution of the FSI (labels) under 0% to 40% REPRs. The number of insecure samples follows an increasing trend with the growth of the REPR. Conversely, the number of absolutely secure samples follows a decreasing trend with the growth of the REPR. Moreover, the number of relatively secure samples approximatively follows a normal distribution. It is assumed that the other operating conditions are not shifted, such that the growth of the REPR deteriorates the frequency stability of power systems.

#### 5.1.1. Feature Subset

**Figure 7:**
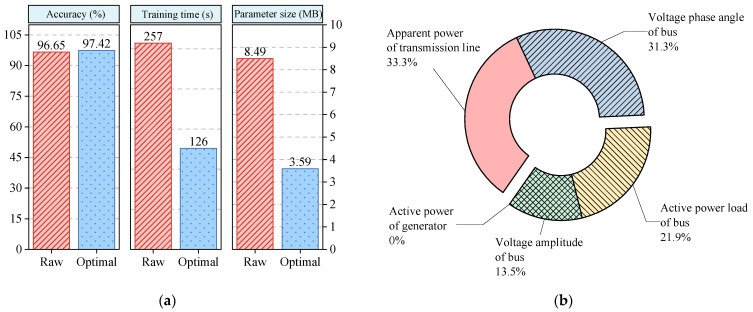
The results of conducting CE-based feature selection on the modified New England 39-bus system: (**a**) comparison between the results obtained on the raw dataset and those obtained on the optimal dataset; (**b**) component analysis of the feature subset with 96 features.

Due to the vast computational resources required by a transformer, a single-layer fully connected network (FCN) is used to verify the effectiveness of CE-based feature selection in this section. Figure 7a depicts a comparison between the raw dataset and the optimal dataset in terms of the accuracy, training time, and parameter size of the FCN. The parameter size denotes the required computational resources, which are mainly dependent on the data dimensionality and model complexity. According to dimensionality reduction, CE-based feature selection can reduce the training time and parameter size while maintaining the original performance as much as possible. Figure 7b shows the component analysis results of the feature subset. In the feature subset, the apparent power of the transmission line accounts for 33.3% of the feature subset, and the voltage phase angle of the bus accounts for 31.3% of the feature subset. This reflects that these physical variables may involve much effective information for the FSP. Subsequently, the active power load accounts for 21.9% of the subset, and the voltage amplitude of the bus accounts for 13.5% of the subset. This reflects that these physical variables only involve some effective information for the FSP. It is worth noting that the active power of the generator accounts for 0%. This reflects that the active power of the generator may provide zero or slight effective information for the FSP. Overall, CE, as a theoretical tool in statistics, tries to analyze the correlation between system frequency and other physical variables of the power system. To some degree, this can help system planners and dispatchers understand what input features are important for the FSP.

Finally, the feature subset provided by CE-based feature selection can substitute for the raw dataset. Thus, the next section adopts this feature subset as the input dataset to compare the performance of different models.

#### 5.1.2. Performance Comparison

A support vector machine (SVM), FCN, LeNet [63], AlexNet [64], ResNet [38], VGG [65], MobileNet [66], and InceptionNet [67] are utilized for comparison to test the performance of the ViT on the same dataset. The SVM and FCN are traditional ML models and are the default implementations provided by Sklearn. LeNet, AlexNet, ResNet, VGG, MobileNet, and InceptionNet are traditional DL models that use the same parameters and structure as those in their original papers. In particular, the structure of the ViT contains three transformer encoder layers and one MLP layer for classification. The detailed hyperparameters of the well-trained ViT model are listed in Table 5. For a fair comparison, the ViT and other DL models adopt the same training strategy.

**Figure 8:**
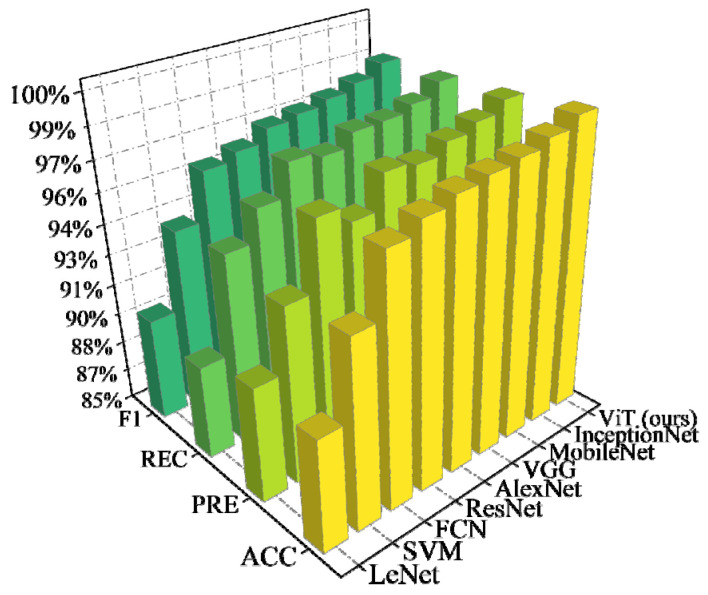
Performance comparison between the ViT and other ML models on the modified New England 39-bus system.

As shown in Figure 8, it is evident that the proposed method achieves SOTA performance in comparison with the traditional ML and DL methods. The *PRE* and *REC* values are similar to the *ACC* values, which reflects that the models treat each FSI category fairly. The traditional ML models lack the powerful feature-extraction capability of DL. Thus, they have poorer performance than the DL models. The traditional DL models extract data information by the convolution layer. However, the convolution layer focuses on the extraction of local data features, and obtaining global data information requires a large number of convolution layers. However, according to the results, MSA is a better effective mechanism than convolution. Unlike convolution, MSA can directly extract global features [35], which leads to better performance for the proposed method. Moreover, since FSP has high demands regarding the execution times of models, the proposed algorithm only takes approximately 0.34 s (the time window is 0.32 s and the execution time is 0.02 s) to be executed for predicting each FSI category since the implementation of transformer models has been highly optimized [36]. Thus, the proposed method is acceptable for online applications.

#### 5.1.3. Influence of Gaussian Noise

The experiments, as mentioned above, are assumed to sample data from the PMUs in power systems without any noise. However, PMUs usually suffer from noise interference and sampling errors [27]. To analyze the influence of white Gaussian noise on the models, this study added a noise *n* with different signal–noise ratios (SNRs) to the feature subset. The SNR is given by Equation (19). The accuracies of the ViT and the other models on the noisy data are reported in Table 6, where the best values are highlighted in boldface.

(19) $${\mathrm{SNR}=10\log}_{10}\frac{\sum_{i=1}^{l}\sum_{j=1}^{h}P_{d}^{2}(i,j)}{\sum_{i=1}^{l}\sum_{j=1}^{h}n^{2}(i,j)},$$

As the SNR declines from 50 dB to 10 dB, Gaussian white noise hinders the useful feature vector information extracted by the ML models, reducing their accuracy. In Table 6, it can be observed that the ViT achieves SOTA accuracy on noisy datasets (from 50 dB to 10 dB) relative to those of other methods. Note that the ViT still exceeds 90% accuracy on the noisy data with an SNR of 10 dB. In contrast, the accuracies of the SVM, the FCN, LeNet, AlexNet, and MobileNet are obviously lower than 90% on noisy data, with an SNR of 10 dB. Overall, the ViT ensures the concentration of useful information in the extracted feature vectors, which illustrates that the ViT can tolerate PMU noise in practice.

#### 5.1.4. Incomplete Data Analysis

Another assumption mentioned above is that the PMU measurements of all data are available. In practice, some PMU data may be missing due to PMU losses or communication delays [27]. To analyze the influences of incomplete data on the models, we randomly set the data of each sample to 0 with the same proportions. The incomplete ratio can be described by Equation (20), where *N*_missing_ is the number of missing data, and *N_all_* is the total number of data. The accuracies of the models on the incomplete data are presented in Table 7, where the best values are also highlighted in boldface.

(20) $$\mathrm{IncompleteRatio}=N_{\mathrm{missing}}/N_{all},$$

As the incomplete ratio rises from 5% to 40%, the missing data also reduce the information contained in the feature vectors extracted by the ML models, which decreases their accuracy. As shown in Table 7, the ViT achieves SOTA accuracy compared with that of other ML methods. Under incomplete ratios of 5% and 10%, the ViT exceeds 95% accuracy. An incomplete ratio of more than 10% is rare in real power systems unless they are maliciously attacked. In the case with malicious attacks, the accuracy of the ViT still remains at 90.78% under an incomplete ratio of 40%. For this situation, one of the reasons for this performance may be attributed to the global feature extraction ability of the transformer model. Additionally, it should be noted that the accuracies of InceptionNet and ResNet are close to that of ViT. The main reason for this may be that stacking a large number of convolution layers can similarly extract global features from local features [38]. However, MSA can naturally extract global features. Thus, the ViT is less affected in terms of performance when data are missing. Finally, the ViT is empirically proven to work more robustly than CNN-based models, even when some PMU measurements are unavailable.

#### 5.1.5. Visualization Analysis of the ViT

**Figure 9:**
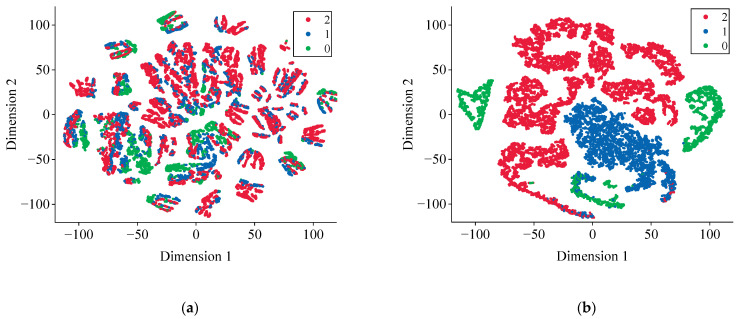
Visualization of the ViT feature extraction results. FSIs from 0 to 2 indicate insecurity, relative security, and absolute security, respectively: (**a**) raw data distribution; (**b**) output of the last layer.

TSNE [68] is a popular method for embedding high-dimensional data to visualize them in a low-dimensional space. To further analyze the representation ability of the ViT, we decrease the dimensions of the feature vectors extracted by the model to 2D for visualization purposes. The results are shown in Figure 9, in which the closer the sample points are, the more similar they are, and the different colors distinguish different categories. Figure 9a shows the feature visualization results obtained from the raw data after performing dimensionality reduction via TSNE, and there are no evident demarcation lines between the three categories. After full training, it is clear from Figure 9b that the chaotic feature is separated into several clusters by the well-trained ViT model. The TSNE visualization results demonstrate the powerful feature extraction ability of the transformer architecture. Moreover, the representations learned by the MSA mechanism are useful for the subsequent classification task. Overall, this proves that the ViT model has the ability to find effective representations for classification.

### 5.2. A Modified ACTIVSg500 System

**Figure 10:**
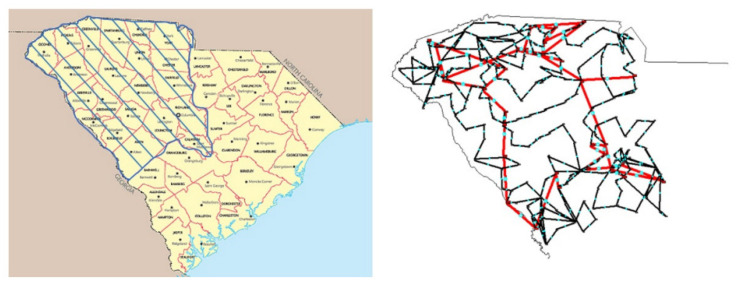
Geographic footprint and one-line diagram of the ACTIVSg500 system.

In addition to the modified New England 39-bus system, a more extensive synthetic system, the modified ACTIVSg500 system, was also employed as a test case to validate the performance and scalability of the proposed method. As shown in Figure 10, the ACTIVSg500 system was built based on the footprint of western South Carolina, covering approximately 21 counties with approximately 2.6 million people [69]. The ACTIVSg500 system has two voltage levels (345/138 kV). Furthermore, it contains 90 generators with a total generation capacity of approximately 12 GW [70]. The synchronous generators include coal, gas, and hydro generators. The nonsynchronous generators include wind and solar PV power plants. Specifically, the detailed parameters of dynamic wind farm models are the same as those in the previous case. The grid interface module for solar generators adopts REGCAU1, the electrical control module for solar generators adopts REECBU1, and the plant controller module for solar generators adopts REPCAU1. The detailed parameters of solar PV power plants are listed in Appendix A. The wind power plants are connected to nodes 9, 144, and 197 of the system, and the solar PV power plants are connected to nodes 17, 167, and 224 of the system. Changing the power outputs of the generating units adjusts the different REPRs.

Similarly, numerical simulations were carried out on PSS/E, and all simulation models were provided by Texas A&M University. The load levels were set to 50%, 52%, …, and 100% of the basic system load levels. The same ratios also scale the generation power but with extra modifications to ensure that all input data stay within a reasonable range. Under each power flow level, the sudden load volatility was set to active power disturbance. The disturbances are located at nodes 4, 6, 61, 64, 103, 150, 204, 292, 303, 364, 470, and 499. The fault sizes were set to range from −700 MW to 700 MW at intervals of 100 MW. The fault occurrence time was set at the simulation start moment (0 s), the simulation duration was 60 s, and the sample rate was 100 Hz for each disturbance. This study set different REPRs: 0%, 5%, …, and 40% of the total generation power output.

The detailed configurations used for dataset generation are summarized in Table 8. The required input data for the FSI calculation process are listed in Table 9. Consequently, 39,312 labeled samples were formed under the above conditions. In further trials, the dataset was also divided randomly at a 7:3 ratio into training and test datasets to assess the observed performance discrepancies.

#### Testing Results and Comparison

**Figure 11:**
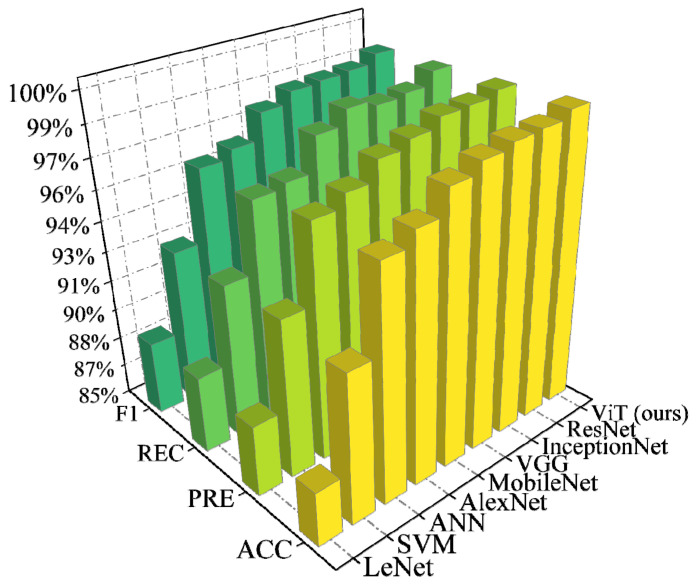
Performance comparison between the ViT and other models on the modified ACTIVSg500 system.

**Figure 12:**
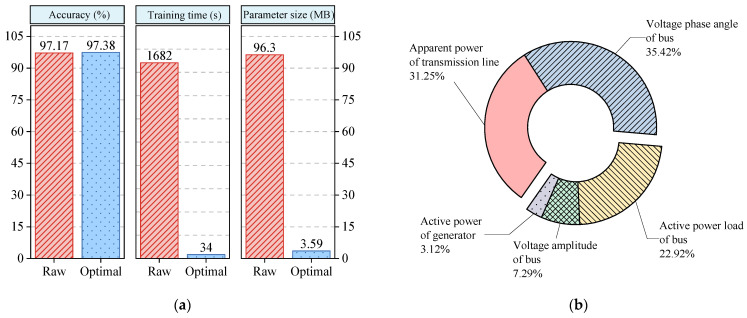
The results of conducting CE-based feature selection on the modified ACTIVSg500 system: (**a**) comparison between the results obtained on the raw dataset and those obtained on the optimal dataset; (**b**) component analysis of the feature subset with 96 features.

The ViT and other methods have almost the same configurations as those in the previous case. The performance comparison between the ViT and other methods is shown in Figure 11. The feature subset of the ACTIVSg500 system was similarly analyzed, and the results are shown in Figure 12. The effects of PMU noise and loss on the model are shown in Table 10 and Table 11, respectively.

In the results presented thus far, this case agrees with the previous case. The proposed ViT-based method achieves the best accuracy among the tested methods, whether they are used in a noisy or incomplete environment or not. This indicates again that the global feature extraction of MSA is better than the local feature extraction of convolution. Note that the performance of the proposed method does not decline as the system size increases, demonstrating its superior efficacy and scalability. The reason behind these results might be that CE retains as many variables as possible that are effective in FSP. In Figure 12b, the variables of power systems follow the same trend as in the previous case, with a slight difference in the number of each feature.

## 6. Discussion

In the present study, we first propose a ViT-based FSP method. Note that the ViT only uses the pure transformer architecture because we aim to explore the potential of the transformer architecture in FSP. Convolution and MSA are all effective mechanisms for extracting useful information. Multimodel combinations may be better when the transformer is reasonably combined with other DL models. For example, the transformer can be combined with a CNN to mine the complex relationships between data because the transformer is good at extracting global features [71] and the CNN is good at extracting local features.

In addition, the transformer is a widespread method in NLP. For NLP, the self-supervised training approach [36] is common; i.e., the training process does not need data labels. However, most ML methods in power systems are supervised training approaches, i.e., the training process needs data labels. The use of self-supervised training methods combined with transformers is foreseeable in power systems. The advantage of self-supervised learning is that it ensures low-cost access to large amounts of training data and maintains high performance. For example, the topology of a power system may change due to a failure, causing the new data to be completely different from the original training data. Models trained via supervised learning cannot handle new data that are outside the range of the original training data. This means that new data should be collected and labeled to retrain the model to fit such a change, but this is a high-cost training approach. In contrast, self-supervised learning can automatically fit the change by collecting data without manual annotation, which costs less than supervised learning.

For the image classification task, DL does not require feature selection because the dimensionality of the image is fixed, and DL can automatically extract useful features. For the FSP of power systems, different power systems have different dimensions, and their data are highly redundant. CE-based feature selection transforms power system operation data into image-like data with three channels and 32 pixels, thereby significantly improving the generalization of the proposed method. Moreover, DL usually belongs to a black-box model that has low interpretability. According to feature selection, we can know feature importance, which increases the interpretability of the resulting model to some extent.

## 7. Conclusions and Future Work

This paper proposes a DL method for power system FSP by using ViT and CE. Case studies were carried out on the modified New England 39-bus system and the modified ACTIVSg500 system. The results demonstrate the following:

- The ViT-based FSP method achieves SOTA performance compared to eight ML methods on normal, noisy, and incomplete datasets, so the proposed method is suitable for practical applications.
- As for the FSP of power systems tasks, the global feature extraction of MSA is a better mechanism than the local feature extraction of convolution.
- When using CE-based feature selection, the proposed method is still efficient and achieves high performance in power systems of any scale without vast computational resources.
- From the point of view of CE, the apparent power of the transmission line and the voltage phase angle of the bus have strong correlations with FSP when the load variance occurs. Conversely, the active power of the generator has a weak correlation with FSP when the load variance occurs.

In the future, the authors hope that this work may extend to modern or future power systems containing all units, i.e., loads, storage devices, and converter-connected units. These results can help system planners and dispatchers make related decisions [72,73] regarding frequency stability and control in power systems.

## Figures and Tables

**Table 1:** SB and IB.

Index (*φ*)	Boundaries	
	SB (*φ*)	IB (*φ*)
Δ*f_c_*	*α* × Δ*f_c_*^max^	Δ*f_c_*^max^
*RoCoF*	*β* × *RoCoF*^max^	*RoCoF* ^max^
Δ*f_s_*	*γ* × Δ*f_s_*^max^	Δ*f_s_*^max^

**Table 2:** Original feature selection.

Number	Original Feature
1	Electrical power of each generator from *t*_0_ to 32 *f*_t_
2	Active power load of each bus from *t*_0_ to 32 *f*_t_
3	Voltage amplitude of each bus from *t*_0_ to 32 *f*_t_
4	Voltage phase angle of each bus from *t*_0_ to 32 *f*_t_
5	Apparent power of each line from *t*_0_ to 32 *f*_t_

Note: *t*_0_ is the initial sampling point when a disturbance occurs. *f*_t_ is the sampling period.

**Table 3:** Configurations used for dataset generation on the modified New England 39-bus system.

Name	Value
Load Levels	50%, 51%, 52%, …, 100%
Fault Buses	3, 4, 7, 8, 12, 15, 16, 18, 20, 21, 23, 24, 25, 26, 27, 28, 29, 31, 39
Fault Sizes (MW)	−500, −400, −300, −200, 200, 300, 400, 500
REPRs	0%, 5%, 10%, 15%, 20%, 25%, 30%, 35%, 40%

**Table 4:** Input data for the FSI calculations of the modified New England 39-bus system.

Disturbance_max_ (MW)	Disturbance_min_ (MW)	Δf_max_ (Hz)	|RoCoF_max_| (Hz/s)	Δfs_des_ (Hz)
±400	±200	0.6	0.5	0.25

**Table 5:** The hyperparameters of the ViT model.

Hyperparameter	Value
Input size	32
Classes	3
Patch size	4
Hidden size	256
Heads	8
MLP size	128
Dropout	0.05

**Table 6:** Test accuracy of different models on the noisy datasets of the modified New England 39-bus system.

Model	Accuracy (%)								
	50 dB	45 dB	40 dB	35 dB	30 dB	25 dB	20 dB	15 dB	10 dB
SVM	93.93	93.81	93.68	93.49	93.02	92.55	91.51	89.05	84.92
FCN	96.36	95.88	94.90	94.13	93.98	93.89	92.16	89.38	85.81
LeNet	89.42	89.33	89.08	88.52	87.16	86.74	85.31	84.95	82.06
AlexNet	97.53	97.29	96.86	96.78	96.63	96.36	94.78	90.23	82.31
InceptionNet	98.16	98.08	97.87	97.39	96.98	96.33	95.22	94.02	90.29
VGG	97.55	97.24	97.07	96.86	96.61	95.91	95.34	93.49	89.16
ResNet	97.27	97.08	96.78	96.58	96.26	95.82	95.04	92.16	90.15
MobileNet	97.81	97.76	97.72	97.35	96.94	96.35	94.24	90.14	81.37
ViT (ours)	**98.86**	**98.54**	**98.39**	**98.21**	**97.97**	**97.42**	**96.56**	**94.79**	**90.94**

**Table 7:** Test accuracies of different models on the incomplete datasets of the modified New England 39-bus system.

Model	Accuracy (%)							
	5%	10%	15%	20%	25%	30%	35%	40%
SVM	87.29	84.44	82.65	80.76	79.16	77.57	76.60	75.72
FCN	87.98	85.08	83.14	81.31	80.55	79.13	78.19	76.93
LeNet	84.49	83.06	82.84	80.88	79.66	79.59	79.28	79.18
AlexNet	92.25	87.62	84.57	79.47	77.61	75.33	73.58	71.44
InceptionNet	96.06	95.29	94.48	93.11	91.79	90.76	89.89	89.78
VGG	94.91	93.04	90.83	90.16	87.92	86.24	85.48	83.82
ResNet	96.63	95.46	94.78	93.78	92.98	91.54	90.49	89.97
MobileNet	87.77	86.27	80.34	76.02	72.08	71.76	69.03	67.76
ViT (ours)	**97.11**	**95.86**	**95.08**	**94.95**	**94.32**	**93.62**	**92.54**	**90.78**

**Table 8:** Configurations used for dataset generation on the modified ACTIVSg500 system.

Name	Value
Load Levels	50%, 52%, 54%, …, 100%
Fault Buses	4, 6, 61, 64, 103, 150, 204, 292, 303, 364, 470, 499
Fault Sizes (MW)	−700, −600, −500, −400, −300, −200, −100, 100, 200, 300, 400, 500, 600, 700
REPRs	0%, 5%, 10%, 15%, 20%, 25%, 30%, 35%, 40%

**Table 9:** Input data used for the FSI calculation process of the modified ACTIVSg500 system.

Disturbance_max_ (MW)	Disturbance_min_ (MW)	Δf_max_ (Hz)	|RoCoF_max_| (Hz/s)	Δfs_des_ (Hz)
±550	±250	1	1	0.4

**Table 10:** Test accuracies of different models on the noisy datasets of the modified ACTIVSg500 system.

Model	Accuracy (%)								
	50 dB	45 dB	40 dB	35 dB	30 dB	25 dB	20 dB	15 dB	10 dB
SVM	92.21	92.18	92.04	91.89	91.54	90.32	89.42	88.21	85.43
FCN	96.65	96.31	96.01	95.87	95.10	94.95	92.27	90.43	87.66
LeNet	88.31	87.47	87.31	86.71	86.98	86.85	86.69	85.71	84.62
AlexNet	97.22	96.52	96.33	96.16	95.23	94.91	92.76	89.62	86.41
InceptionNet	98.63	98.53	98.48	98.08	97.79	95.41	93.82	91.39	88.99
VGG	98.82	98.57	98.55	98.31	97.86	96.33	94.12	90.54	88.38
ResNet	98.94	98.69	98.48	97.94	97.29	95.49	93.13	90.97	88.49
MobileNet	98.96	98.68	98.30	97.09	95.28	92.83	90.89	88.92	85.17
ViT (ours)	**99.12**	**99.04**	**98.96**	**98.48**	**98.37**	**97.47**	**95.46**	**91.97**	**89.55**

**Table 11:** Test accuracies of different models on the incomplete datasets of the modified ACTIVSg500 system.

Model	Accuracy (%)							
	5%	10%	15%	20%	25%	30%	35%	40%
SVM	90.78	90.16	89.73	89.02	88.76	88.23	87.75	87.36
FCN	89.55	88.09	86.69	86.47	86.02	85.67	84.95	84.57
LeNet	85.21	84.95	84.00	83.57	82.75	82.66	81.97	81.89
AlexNet	91.59	88.88	86.87	86.71	85.33	85.04	84.25	83.69
InceptionNet	94.03	91.11	90.81	90.12	89.85	88.99	88.32	87.87
VGG	92.73	91.45	90.27	89.84	88.84	88.26	87.47	87.18
ResNet	94.47	92.17	91.11	90.24	89.32	88.66	88.13	87.70
MobileNet	89.77	88.35	86.87	85.72	85.23	84.25	83.42	83.25
ViT (ours)	**95.04**	**93.23**	**92.74**	**91.27**	**90.95**	**90.36**	**89.98**	**89.52**

## Data Availability

Not applicable.

## Appendix A

The detailed parameters of dynamic wind farm models are listed in Table A1, Table A2, Table A3 and Table A4. The detailed parameters of solar PV power plants are listed in Table A5, Table A6 and Table A7.

**Table A1:** The parameters of WT3G2 module.

**#1**	**#2**	**#3**	**#4**	**#5**	**#6**	**#7**
0.20	0.0	0.0	0.0	0.10	1.50	0.50
**#8**	**#9**	**#10**	**#11**	**#12**	**#13**	
0.90	1.0	1.20	2.0	5.0	0.02	

**Table A2:** The parameters of WT3T1 module.

**#1**	**#2**	**#3**	**#4**	**#5**	**#6**	**#7**	**#8**
1.25	4.95	0.0	0.7 × 10^−2^	21.98	0.0	1.8	1.5

**Table A3:** The parameters of WT3P1 module.

**#1**	**#2**	**#3**	**#4**	**#5**	**#6**	**#7**	**#8**	**#9**
0.30	150.0	25.0	3.0	30.0	0.0	27.0	10.0	1.0

**Table A4:** The parameters of WT3E1 module.

**#1**	**#2**	**#3**	**#4**	**#5**	**#6**	**#7**	**#8**
0.15	18.0	5.0	0.0	0.05	3.0	0.60	1.12
**#9**	**#10**	**#11**	**#12**	**#13**	**#14**	**#15**	**#16**
0.10	0.296	−0.436	1.10	0.05	0.45	−0.45	5.0
**#17**	**#18**	**#19**	**#20**	**#21**	**#22**	**#23**	**#24**
0.05	0.90	1.20	40.0	−0.50	0.40	0.05	0.05
**#25**	**#26**	**#27**	**#28**	**#29**	**#30**	**#31**	
1.0	0.69	0.78	0.98	1.12	0.74	1.20	

**Table A5:** The parameters of REGCAU1 module.

**#1**	**#2**	**#3**	**#4**	**#5**	**#6**	**#7**
0.2 × 10^−1^	10.0	0.90	0.50	1.22	1.20	0.80
**#8**	**#9**	**#10**	**#11**	**#12**	**#13**	**#14**
0.40	−1.30	0.2 × 10^−1^	0.70	9999.0	−9999.0	1.0

**Table A6:** The parameters of REECBU1 module.

**#1**	**#2**	**#3**	**#4**	**#5**	**#6**	**#7**	**#8**	**#9**
−99.0	99.0	0.0	−0.5 × 10^−1^	0.5e-0.1	0.0	1.05	−1.05	0.0
**#10**	**#11**	**#12**	**#13**	**#14**	**#15**	**#16**	**#17**	**#18**
0.5 × 10^−1^	0.436	−0.436	1.10	0.90	0.0	0.10	0.0	40.0
**#19**	**#20**	**#21**	**#22**	**#23**	**#24**	**#25**		
0.2 × 10^−1^	99.0	−99.0	1.0	0.0	1.82	0.2 × 10^−1^		

**Table A7:** The parameters of REPCAU1 module.

**#1**	**#2**	**#3**	**#4**	**#5**	**#6**	**#7**	**#8**	**#9**
0.2 × 10^−1^	18.0	5.0	0.0	0.75 × 10^−1^	0.0	0.0	0.0	0.2 × 10^−1^
**#10**	**#11**	**#12**	**#13**	**#14**	**#15**	**#16**	**#17**	**#18**
0.10	−0.10	0.0	0.0	0.436	−0.436	0.10	0.5 × 10^−1^	0.25
**#19**	**#20**	**#21**	**#22**	**#23**	**#24**	**#25**	**#26**	**#27**
0.0	0.0	999.0	−999.0	999.0	−999.0	0.10	20.0	0.0
